# Research progress in the inhibitory mechanisms of traditional Chinese medicine therapies against influenza A

**DOI:** 10.3389/fimmu.2026.1764099

**Published:** 2026-05-05

**Authors:** Mingjiang Liu, Shitong Han, Ninghao Shi, Tao Zhang, Chenglong Yu, Chen Mei, Xiaolong Xu

**Affiliations:** 1 College of Veterinary Medicine, Jiangsu Co-innovation Center for Prevention and Control of Important Animal Infectious Diseases and Zoonoses, Yangzhou University, Yangzhou, China; 2 Jiangsu Co-innovation Center for Prevention and Control of Important Animal Infectious Diseases and Zoonoses, Yangzhou, China; 3 Institute of Animal Husbandry and Veterinary Medicine, Beijing Academy of Agriculture and Forestry Sciences, Beijing, China; 4 Beijing Hospital of Traditional Chinese Medicine, Capital Medical University, Beijing, China

**Keywords:** antiviral activity, immunomodulation, influenza A, inhibition mechanism, traditional Chinese medicine

## Abstract

Influenza A, an acute or subacute respiratory infectious disease caused by the influenza A virus (IAV), affects humans and animals, and poses a serious threat to public health. Currently, IAV prevention and treatment rely primarily on antiviral drugs and nebulization therapy. Although these methods offer rapid efficacy, prolonged or excessive use often induces drug resistance and sequelae, and has high costs. Throughout China’s history, traditional Chinese medicine (TCM) has repeatedly resisted major epidemics, and substantially contributed to the survival and cultural continuity of the Chinese nation. Modern medical research has demonstrated that certain traditional Chinese medicinal formulas or their primary active constituents exhibit significant antiviral activity and immunomodulatory effects. Consequently, exploring the antiviral mechanisms of TCM or their active components, alongside their synergistic application with influenza vaccines and existing antiviral drugs, could enable novel approaches and research directions to achieve influenza prevention and control. This article reviews the current status of IAV transmission, as well as progress in modern medical research in influenza A prevention and treatment with TCMs or their active constituents.

## 1 Introduction

Influenza A virus (IAV), a pathogen causing acute respiratory infections, is characterized by high transmissibility and widespread prevalence, particularly during the winter and spring seasons. Common clinical symptoms include high fever, cough, sore throat, and muscle pain. Severe cases may progress to pneumonia or multiple organ failure, and can even become life-threatening. In recent years, global climate change and human activities have contributed to rising influenza A incidence rates, which in turn have posed substantial public health challenges. Traditional Chinese medicine (TCM) has a long history of understanding influenza A. The ancient text *Treatise on Epidemic Fevers* contains records of the high transmissibility of influenza A, which was believed to spread through respiratory, digestive, and conjunctival routes, thus leading to similar symptom presentations. TCM has accumulated extensive experience in influenza A prevention and treatment, emphasizing holistic concepts and syndrome differentiation. This approach alleviates symptoms and decreases complication rates by regulating the body’s immune function. Because Chinese herbal components not only exert multi-targeted, multi-pathway regulatory effects but also influence the viral life cycle, they have promise in achieving preventive and therapeutic outcomes.

## 2 Methods

This is a narrative review aimed at systematically summarizing the research progress on the mechanism of action of TCM against IAV. To improve the transparency and reproducibility of the review, the literature identification and screening methods are briefly described below:

### 2.1 Literature search:

Relevant literature was collected by searching Chinese and English databases such as PubMed, Web of Science, CNKI, and Wanfang Data Knowledge Service Platform. The search period mainly covered 2010 to 2026.

### 2.2 Search strategy

A combination of subject terms and free terms was used for searching. English search terms included “TCM”, “Chinese herbal medicine”, “influenza A”, “H1N1”, “antiviral”, “immunomodulation” and “mechanism”. Chinese search terms included “TCM”, “influenza A”, “H1N1”, “antiviral”, “immunomodulation”, and “mechanism”.

### 2.3 Inclusion and exclusion criteria

Inclusion criteria included *in vitro*, animal, and clinical studies exploring the anti-IAV mechanisms of action (including direct antiviral, immunomodulatory, anti-inflammatory, and metabolic regulation) of TCM compound formulas, single herbs, or their active ingredients. Exclusion criteria were: 1) Non-Chinese/English literature; 2) Literature describing only clinical efficacy without exploring the mechanism; 3) Literature clearly unrelated to the topic; 4) Duplicate publications or literature for which full text was unavailable.

## 3 Results and discussion

### 3.1 Current trend of popularity

In exploring influenza prevention and control strategies, understanding the viral origins and mechanisms of cross-species transmission is crucial. The primary natural hosts of IAVs are waterfowl. Because avian IAVs have not only breached interspecies barriers but also triggered epizootics and epidemics in various mammals, they pose a severe threat to animal health (1). Although swine flu itself has a relatively low mortality rate, it is frequently found in co-infections with other pathogens, thus significantly increasing mortality rates in pig populations and causing substantial losses to the livestock industry (2). A study by Shaikh et al. across ten provinces in China has identified a novel IAV in pig populations called genotype 4 (G4) reassortant Eurasian avian-like H1N1 virus (G4 EA H1N1). The detection of antibodies to this virus in humans exposed to infected pigs has indicated its strong potential for human infection. The virus carries genetic fragments from the 2009 H1N1 pandemic virus, exhibits high binding affinity toward human respiratory cell receptors, and replicates efficiently in human bronchial and alveolar epithelial cells, thereby demonstrating substantial pathogenicity and transmission potential (3). Most people who contract the flu experience sudden respiratory symptoms and muscle aches, with or without fever, and recover within a week. However, some may develop serious or fatal complications. According to the World Health Organization, 3 to 5 million people are infected with the IAV each year, resulting in 290,000 to 650,000 deaths globally (4, 5). Other viruses, such as influenza A and B, as well as seasonal coronaviruses, also significantly increase the annual burden of respiratory illness. Vulnerable populations, including the elderly, young children, and those with weakened immune systems, face particularly high risks (6). This underscores the urgent need for effective and widely available antiviral treatments (7).

Beyond pigs, companion dogs have also become important monitoring targets. Lyu et al. detected the canine influenza virus (CIV) H3N2 subtype in 13.5% of 399 canine respiratory samples collected from multiple regions in China between 2012 and 2017. Phylogenetic analysis revealed that strains isolated after 2016 formed an independent branch closely related to strains from South Korea and the United States. These findings suggest possible introduction into China through one or multiple events in 2016. These strains appear to have strong interspecies adaptability, because they exhibit key amino acid mutations similar to those in human IAVs. The unique antigenic characteristics in post-2016 strains, revealed through antigenic analysis, suggest that ongoing antigenic drift might compromise vaccine efficacy (8). Ratanaporn et al. have confirmed the efficient transmission and pathogenicity of Thai CIV-H3N2 in dogs through infection experiments, in which both vaccinated and directly exposed dogs exhibited symptoms including fever, depression, elevated nasal and ocular discharge, coughing, and substantial respiratory viral shedding (9). The threat of canine influenza A extends beyond its transmissibility. Tangwangvivat et al. have noted that canine IAVs comprise primarily two subtypes: H3N8, derived from equine IAVs, and H3N2, originating from avian IAVs. Both have persisted in canine populations across North America, Asia, and Europe, and new strains have been generated through genetic reassortment. More critically, CIVs have substantial interspecies transmission potential and can infect other mammals beyond dogs, including cats. Because large populations of dogs are companion animals with close human contact, dogs might serve as “mixing vessels” for genetic recombination among avian, human, and canine IAVs, thus increasing the likelihood of emergence of novel IAVs with potential zoonotic risks (10).

Cats’ susceptibility to IAVs also warrants attention. A review by Frymus et al. has indicated that, despite historical assumptions of feline resistance to influenza A, cats have contracted multiple IAV subtypes originating from other species. Low-pathogenic IAV infections in cats typically manifest subclinical or mild respiratory symptoms, and severe cases occur predominantly in densely populated environments such as animal shelters. For instance, canine-derived H3N2 has been found to cause as much as 100% morbidity and 40% mortality in shelter cat populations. Additionally, cats can be infected with avian-derived H7N2. The highly pathogenic avian-derived H5N1 virus responsible for the 2009 human pandemic can cause severe systemic disease and has been found to be transmitted among cats (11). Since the identification of the H5N1 virus, its evolution has spawned multiple branches and sub-branches. In early 2024, HPAI-H5N1 evolutionary line 2.3.4.4b began circulating in Texas dairy cows (12).

Overall, IAV infections in carnivores such as dogs and cats originate primarily from direct spillover of avian IAVs or infection with human or swine IAVs. Among dogs, H3N8 (equine origin) and H3N2 (avian origin) viruses have caused sustained epidemics in various regions since 1999 and 2004, respectively, and enhanced transmission efficiency has been observed in high-density canine settings (e.g., shelters or boarding facilities). Cats experienced an outbreak of H7N2 avian influenza A with enhanced adaptability in a New York shelter in 2016. Although dogs and cats exhibit relatively low susceptibility to human IAVs, and no large-scale influenza A outbreaks have been recorded in dogs, the repeated interspecies transmission of viruses and their close contact with humans suggest that these companion animals might pose potential public health risks by acting as viral “mixing vessels” (13, 14). In April 2022, China reported its first case of human infection with H3N8 in Henan Province, followed by other cases in Hunan Province (15–18). Sequence analysis showed that the isolated virus had the highest homology with avian H3N8 virus, indicating sporadic cross-species transmission from birds to humans (15, 18).

Currently, the medical community widely uses targeted antiviral drugs and antibiotics to treat influenza A. However, these medications are costly, highly toxic, and prone to drug resistance (19, 20). According to TCM theory, the disease is mainly located in the lungs and spleen. The basic pathogenesis is “dampness, toxins, heat, phlegm, plasma and deficiency” (21). Early clinical manifestations of the disease may include fever or no fever. Fever is often accompanied by a variety of symptoms, such as latent fever, dry cough, fatigue, vomiting, loose stools, diarrhea and other digestive symptoms. The tongue is usually greasy and has obvious dampness and toxicity due to the disease. Throughout the clinical process, attention must be paid to eliminating dampness, eliminating turbidity, eliminating dirt and removing toxicity, as well as the effect of blood stasis (22). TCM has been used to treat epidemics since ancient times and has accumulated rich successful experience over thousands of years (22, 23). The advantage of TCM is that even if the cause is unknown, it can propose corresponding prescriptions based on the theory of syndrome differentiation and treatment, based on clinical symptoms, thereby relieving clinical symptoms, shortening the course of the disease and preventing the disease from worsening. A host cell infected by IAV, surrounded by six thematic modules arranged in a circular or grid pattern. The modules depict the key mechanisms by which TCM combats influenza A.

**Figure 1:**
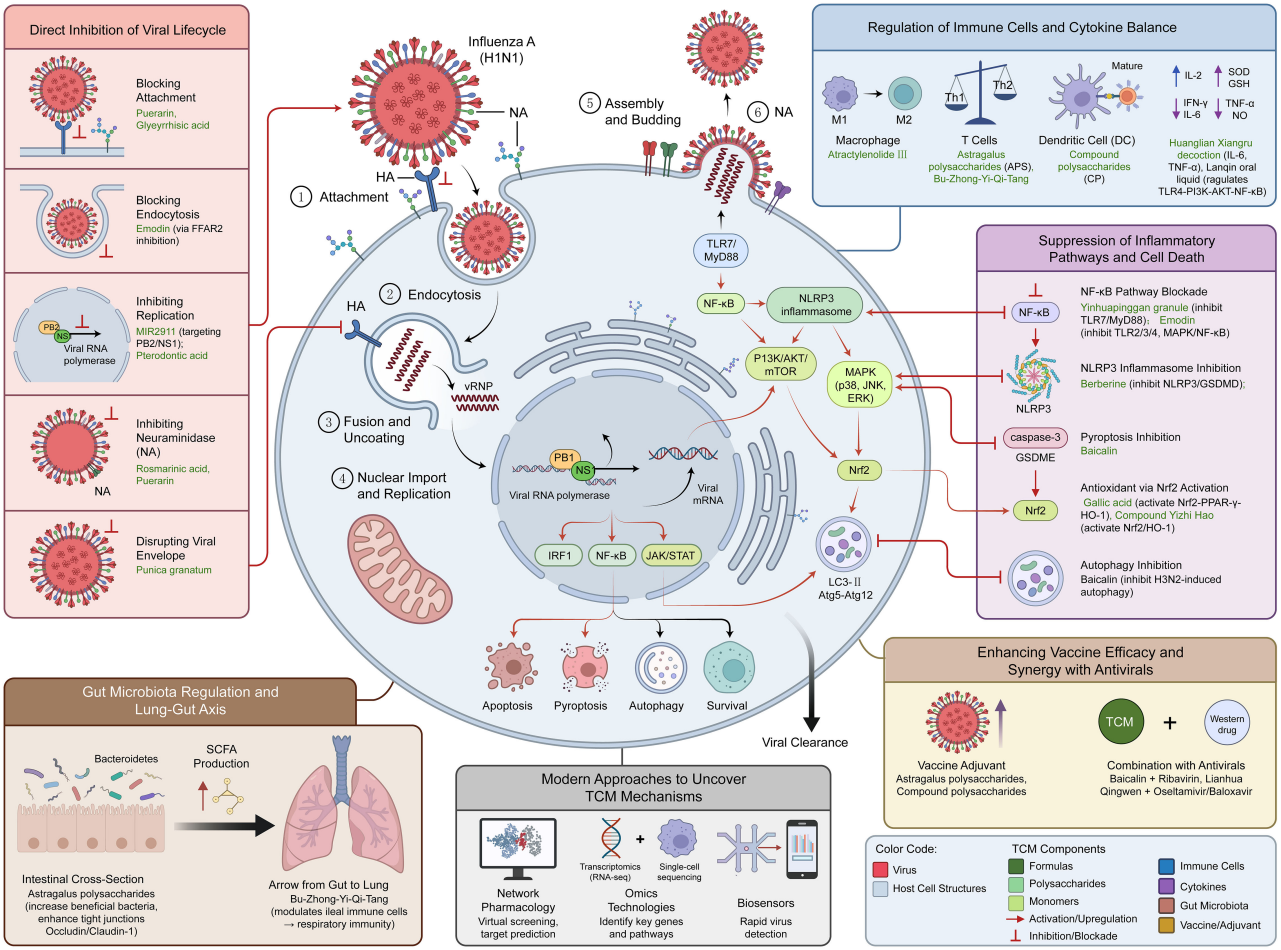
Schematic diagram of IAV transmission and mechanisms of action of TCM therapies against IAV.

In contrast, TCM, which uses natural herbal ingredients, offers lower toxicity and susceptibility to drug resistance, and provides an alternative approach to influenza A prevention and treatment. In-depth research on the mechanisms of action of TCM therapies against influenza A not only provides scientific support for TCM-based influenza therapy but also offers novel insights for developing new antiviral drugs. These findings have advanced the modernization of TCM, promoted the integration of Chinese and Western medicine, and enhanced the effectiveness of influenza A prevention and treatment. A schematic diagram of TCM treatments for influenza A is shown in **Figure 1**.

A host cell infected by IAV, surrounded by six thematic modules arranged in a circular or grid pattern. The modules depict the key mechanisms by which TCM combats influenza A.

### 3.2 Inhibitory effects of TCM therapies against influenza A

#### 3.2.1 Antiviral and anti-inflammatory activity of TCM

Multiple TCM formulations effectively mitigate pulmonary inflammatory damage caused by IAV infection by suppressing key inflammatory factor expression, modulating immune responses, and directly inhibiting viral replication. For instance, the active constituents of Yin Hua Ping Gan granules (including ephedrine hydrochloride, pseudoephedrine, and chlorogenic acid) have been found to significantly inhibit the activation of the Toll-like receptors 7/myeloid differentiation primary response gene 88 (TLR7/MyD88) signaling pathway in canine renal epithelial cells (MDCK) induced by A/Puerto Rico/8/1934(H1N1). Subsequent decreases in mRNA expression of the downstream genes TRAF6, JNK, p38, MAPK, and p65 lead to diminished secretion of interferon-β (IFN-β) and interleukin-6 (IL-6) (24). Nebulized inhalation of Hotoxin solution has been demonstrated to significantly decrease the lung index in PR8-infected mice, alleviate pathological damage to lung tissue, lower the viral load, and decrease IL-6 and tumor necrosis factor-α (TNF-α) levels (25). In the lung tissue in mice infected with IAV, Qingjie Fanggan granules have been found to alleviate pathological changes such as vascular congestion, serous exudation, inflammatory cell infiltration, hyaline membrane formation, and fibrous proliferation. This treatment decreases the lung index, lesion index, and mortality, and protects against lung damage and death (26). Shufeng Jiedu capsules have been demonstrated to inhibit the autophagy marker Microtubule-associated protein light chain 3 II (LC3-II) in the serum of H1N1-infected mice, and to significantly decrease viral nuclear protein (NP) protein expression, M1 gene expression, and interleukin-1β (IL-1β) and TNF-α levels (27). Kudzu root decoction not only inhibits adsorption of the H1N1 virus but also exhibits anti-inflammatory and immunomodulatory activity. This treatment has been found to significantly decrease the expression of proinflammatory factors (IL-1α, IL-6, TNF-α) in lung tissue of non-lethally infected mice, and to normalize the CD4^+^IFN-γ^+^/CD4^+^IL-4^+^ ratio in peripheral blood lymphocytes and TH1/TH2 imbalances (28). The mechanisms of action of TCM compound formulations against influenza A are shown in **Table 1**.

**Table 1:** Mechanisms of action of TCM compound formulations against influenza A.

Number	Traditional chinese medicine	Active ingredient	Influenza virus	Signaling pathway	Experimental subject	Inflammation regulation	Reference
1	Yinhuapinggan Granule	ephedrine hydrochloride, pseudoephedrine hydrochloride, chlorogenic acid, and emodin	H1N1	Inhibition of TLR7/MyD88 signaling pathway activation, Downregulation of mRNA expression in TLR7, MyD88, TRAF6, JNK, p38, MAPK, and p65.	MDCK cell	Reduce IFN-β and IL-6 secretion	(24)
2	Reduning	Wild Honeysuckle Flower, Weeping Forsythia Capsule, Natural Indigo	H1N1	NLRP3-Caspase1	Balb/c mice	Reduced lung index in infected mice, alleviated pathological damage to lung tissue, decreased viral load, and reduced levels of IL-6 and TNF-α.	(25)
3	Qingjie Antiviral Granules	Fineleaf Schizonepeta Herb, Peucedani Radix, liquorice root	H1N1	Nucleoprotein (NP protein) is significantly reduced, and the viral M1 gene is significantly reduced.	Balb/c mice	Downregulation of IL-1β, IL-6, and TNF-α expression	(26)
4	Shufeng Jiedu Capsules	Giant Knotweed Rhizome, Weeping Forsythia Capsule, Indigowoad Root, Chinese Thorowax Root	H1N1	Downregulate the expression of viral NP protein and M1 gene	RAW264.7 cell line	Reduce the levels of IL-1β and TNF-α	(27)
5	Kudzu Root Decoction	Lobed Kudzuvine Root, Chinese Ephedrs Herb, Cmnamomi Mmulus, Rhizoma Alpiniae Galangae, liquorice root, red paeony root, Jujube	H1N1	Targeting the inhibition of NA, the amino acid residues near the NA active site (such as Arg-152, Arg-224, Glu-227, and Asp-151) play an important role in the binding of puerarin to NA.	MDCK cell line, BALB/c mice	Pathological section results: reduced inflammatory response in the lungs of mice	(28)
6	Bu Zhong Yi Qi Tang	Indigowoad Root, Chinese Angelica, Largetrifoliolious Bugbane Rhizome	Not mentioned	Not mentioned	BALB/c mice	Promote the aggregation of lymph T lymphocytes to produce IL-4 and IFN-γ	(29)
7	Compound polysaccharides extracted from shiitake mycelium, Poria, and Tremella	Compound polysaccharides	H1N1	CP-40 synergistically increases IAV-specific IgG levels in serum, thereby reducing the attack of influenza virus on mice	BALB/c mice	Significantly increase serum anti-IAV antibody titers and the concentrations of leukocyte interleukins IL-2, IL-5, and IL-6	(30)
8	Jinhua Qinggan Granules, Lianhua Qingwen Capsules, Qingfei Paidu Decoction, Xuanfei Baidu Granules, Huashi Baidu Granules, and Xuebijing Injection	Quercetin, flavonoids, baicalin, and glycyrrhizic acid	COVID-19, H1N1, H7N9, B influenza virus, dengue virus, and Ebola virus	Target to ACE 2, 3CL protein and IL-6	Human	Regulate IL-6, IL-10 and TNF-α expression	(23)
9	FuFangYiZhiHaoKeLi	Shortstalk Monkshood Root, Iscais indigotica Fortune, Indigowoad Leaf	H1N1, H3 N2	Activate the Nrf2/HO-1 signaling pathway	MDCK cell line	Upregulate the expression of HO-1 mRNA and protein	(31)
10	Huanglian Xiangru Decoction	Mosla Chinensis Maxim, Coptis Root, Officinal magnolia bark	H1N1	Enhance the body’s antioxidant capacity, regulate immune function, and modulate host TLRs pathways	BALB/c mice	Significantly reduced the levels of IL-6, TNF-α, IFN-γ, and NO in the serum of infected mice, and significantly increased the levels of IL-2, SOD, and GSH.	(32)
11	LanQinKouFuYe	Indigowoad Root, baical skullcap root, Common Gardenia Fruit, Amur Cork-tree Bark, Boat - fruited Sterculia Seed	H1N1	Regulate the TLR4-PI3K-AKT-NF-κB signaling pathway	BALB/c mice	Reduce the content of inflammatory factors in bronchoalveolar lavage fluid	(33)
12	FuFangShuangHuaKouFuYe	Wild Honeysuckle Flower, Weeping Forsythia Capsule, Common Andrographis Herb, Indigowoad Root	H1N1	IFN signaling pathway and NLRP3 inflammatory signaling pathway	Mice	Downregulate inflammatory factors TNF-α, CXCL1, IL-18/6/1β, CCL-2/5/10, IFN-α/β/γ, OAS1/3, IRF1, and IFNB1	(34)

#### 3.2.2 Immunomodulatory effects of polysaccharides from TCMs

TCM plays an essential role in combating viral infections by enhancing the body’s immunity. TCM has been demonstrated to promote immune cell function through multiple pathways, thereby increasing the body’s resistance to pathogens. As key immunomodulatory components, polysaccharides in TCM activate immune responses by binding receptors on immune cell surfaces and consequently strengthen the body’s defenses. Diseases affecting the digestive and respiratory tracts, as well as weakness after critical illness, have substantial immune system involvement. Multiple components in the Bu Zhong Yi Qi Tang formula synergistically act on immune-active cells in the mesenteric lymph nodes, thereby regulating the lower respiratory tract immune system (29).

Zhao et al. have demonstrated that Astragalus polysaccharides (APS), when used as adjuvants in influenza A split virus vaccine and recombinant SARS-CoV-2 vaccine, significantly enhance vaccine-induced antibody levels. In lethal IAV challenges, this treatment has been found to exhibit robust protective effects by increasing mouse survival rates and mitigating weight loss. APS achieves bidirectional immune modulation by promoting lymphocyte proliferation and cytokine secretion when antibody levels are low, and by moderately suppressing inflammatory factors when antibody levels are high, thereby maintaining immune equilibrium. RNA sequencing has revealed that the nuclear transcription factor-κB (NF-κB) signaling pathway and Fcγ receptor-mediated phagocytosis play critical roles in APS-enhanced recombinant SARS-CoV-2 vaccine immune responses (35).

Wan et al. have systematically evaluated the immunopotentiating effects of APS as an adjuvant for the H1N1 influenza A vaccine and its regulatory role on the gut microbiota. APS significantly enhances vaccine-induced IgG antibody levels and neutralizing antibody titers, while increasing the proportion of peripheral blood CD8^+^ T cells. Furthermore, APS increases gut microbiota diversity and abundance, markedly alters dominant bacterial communities, enhances the intestinal architecture, and promotes the expression of tight junction proteins (Occludin and Claudin-1). APS has also been reported to strengthen chicks’ resistance to lethal viral infection, significantly decrease serum inflammatory cytokine TNF-α levels, and mitigate alveolar damage (36).

Beyond monosaccharides, complex polysaccharides (CP) also have potential as vaccine adjuvants. Zhang et al. have investigated the immunopotentiating effects of CP and their subcomponents extracted from shiitake mushroom mycelium, poria cocos, and white fungus on inactivated influenza A vaccines. In animal studies, CP have been found to significantly increase mouse survival rates and levels of serum-specific IgG and cytokines (such as IL-2 and IL-5), and to decrease viral load in the lungs and weight loss. These polysaccharides modulate the Th1/Th2 balance, suppress excessive inflammation (e.g., by decreasing IFN-γ), and mitigate pathological lung damage (30).

In summary, polysaccharide components in TCM significantly enhance the body’s immunity through multiple mechanisms, including activating immune cells, regulating cytokines, bidirectionally modulating the immune balance, protecting barrier functions, and regulating the gut microbiota. These findings provide robust support for combating viral infections. With advances in research on the immunomodulatory mechanisms of TCM, potential applications of more herbal components are expected to be uncovered, thus opening new avenues for clinical antiviral treatments and vaccine development. The mechanisms of single Chinese medicinal compounds against influenza A are shown in **Table 2**.

**Table 2:** Mechanisms of action of Chinese herbal monomers against influenza A.

Number	Active ingredients in TCM	IAV	Pathway	Experimental subject	Inflammation regulation	References
1	Astragalus polysaccharide	H1N1	Regulate Th1 and Th2 immunity.	BALB/c mice	APS has bidirectional regulatory characteristics; it upregulates the expression levels of intestinal tight junction proteins Occludin and Claudin-1, and decreases the expression level of serum TNF-α.	(35, 36)
2	Astragaloside and hesperidin	H1N1; human respiratory syncytial virus (HRSV A2)	Inhibiting the NF-κB pathway reduces the expression of ICAM-1	Human Non-Small Cell Lung Cancer Cells (A549)	Alleviated virus-induced lung injury	(37)
3	Chrysophanol	H1N1	Inhibit TLR2, TLR3, TLR4 and downstream Akt, p38, JNK, MAPK, and NF-κB signaling pathways	MDCK, A549 cell line, BALB/c mice	Reduce the expression of IL-1β, IL-6, IL-8, TNF-α, MMP2, MMP3, and MMP9	(38)
4	Astragalus decoction is rich in a specific plant small RNA molecule, MIR2911.	H1N1, H3N2, H5N1 and H7N9	Inhibiting the expression of viral proteins PB2 and NS1 significantly reduces viral replication efficiency.	BALB/c mice	Not related to the content	(39)
5	Eupatorium adenophorum acid	H1N1, H3N2	Inhibit the NF-κB signaling pathway	MDCK, A549 cell line	Inhibit the expression of IL-6, IP-10, MIP-1β, MCP-1, and TNF-α	(40)
6	Sage phenol	H1N1, H3N2, H5N1, H7N9	Directly destroying the viral envelope leads to virus inactivation, as well as inhibiting the expression of proteins and mRNA in the early stages of the viral lifecycle, and suppressing the Jak2/STAT3 signaling pathway	BALB/c mice	Alleviate pneumonia symptoms in mice	(41)
7	Baicalein and baicalin	H3N2	Inhibit virus-induced cellular autophagy	A549, Ana-1 cell line	Not related to the content	(42)
8	Berberine	H1N1	Inhibit the NLRP3/GSDMD pathway	J774A.1 cell line	Inhibit the release of IL-1β and TNF-α	(43)
9	Atractylenolide	H1N1	Downregulate PI3K/Akt pathway	MLE-12 cells, MDCK, A549, BEAS-2 B cell line, C57 BL/6 mice	Downregulate IFN-1 pro-inflammatory signaling	(44)
10	Gallic acid	H1N1, H3N2, H9N2, H7N3	Nrf 2-PPAR-γ-HO-1	MDCK cell line	Reduce IL-6, IL-8, IP-10, MCP-1, RANTES, and TNF-α	(45)
11	Epigoitrin	H1N1	Downregulate MFN2 and upregulate MAVS expression	Kunming mice	Increase the production of IFN-β and IFITM3	(46)

### 3.3 Research progress in mechanisms of TCM therapies in inhibiting influenza A

#### 3.3.1 Directly counteracting IAV by interfering with viral invasion and replication

Viral entry into cells generally involves six steps: adsorption, penetration, uncoating, biosynthesis, assembly, and release. Viruses bind specific receptors on host cells via their surface glycoproteins. Subsequently, the viral envelope fuses with the cell plasma membrane and releases the viral ribonucleoprotein complex (RNP) into the cytoplasm. During transcription, the viral RNA is synthesized, and full-length antisense RNA is produced through replication, thus serving as a template for synthesis of viral genomic RNA. The newly synthesized RNP complex assembles with viral structural proteins at the cell membrane or Golgi membrane, and newly synthesized progeny viruses are subsequently released. TCM exerts antiviral effects primarily by blocking viral adsorption or inhibiting viral replication.

##### 3.3.1.1 Preventing virus adsorption and penetration

The hemagglutinin (HA) on the surface of the IAV capsid is immunogenic. HA can be hydrolyzed by the host into a light chain and a heavy chain. The heavy chain binds sialic acid receptors on the host cell membrane, whereas the light chain facilitates fusion between the viral envelope and the host cell membrane, and enables the injection of viral genetic material to infect cells. HA comprises 16 distinct molecular variants that enable viral particles to attach to cell surfaces, whereas neuraminidase (NA) consists of nine molecular variants that digest host secretions and subsequently release viral particles into host cells (47). The initial step of infection involves viral adsorption to, and penetration into, host cells. The creation of pores on the host cell surface that render the cell membrane brittle and susceptible to deformation and rupture facilitates viral particle release. Intercellular cell adhesion molecule-1 (ICAM-1), a cell surface glycoprotein and adhesion receptor, also transmits biochemical signals. This glycoprotein regulates viral copy numbers during early infection stages, activates NF-κB proteins, and upregulates the NF-κB signaling pathway (48).

Viral genomes (vRNPs) participate in most major subnuclear structures, and use host chromatin, transcriptional mechanisms, and the splicing apparatus to achieve efficient viral mRNA synthesis and export (49). Liu has found that modified Yupingfeng San (Jiawei Yupingfeng San) decreases ICAM-1 expression in a dose-dependent manner at both the mRNA and protein levels. Its extract, glucosyl-Chenpi-Gan, blocks IAV entry and release by inhibiting viral sialinase, whereas astragaloside IAV and naringin decrease ICAM-1 expression by suppressing the NF-κB pathway (37). Wang has demonstrated that emodin acts primarily by blocking viral adsorption and replication processes rather than by directly inactivating the virus. Dihydrocinnamaldehyde demonstrates potent antioxidant ability, by effectively alleviating IAV-induced oxidative stress through decreasing intracellular Malondialdehyde (MDA), NO, and reactive oxygen species levels, while increasing Glutathione (GSH) content and the activity of multiple antioxidant enzymes. Furthermore, emodin regulates immune inflammatory responses, by inhibiting IAV-activated TLR2, TLR3, and TLR4, as well as downstream protein kinase B (Akt), p38, c-Jun N-terminal kinase (JNK), Mitogen-Activated Protein Kinase (MAPK), and NF-κB signaling pathways, and additionally decreasing the expression of proinflammatory cytokines (IL-1β, IL-6, IL-8, and TNF-α) and matrix metalloproteinases (MMP2, MMP3, and MMP9) (38). Aqueous extract of Pueraria root decoction does not influence the penetration phase of viral infection but inhibits viral adsorption in a dose-dependent manner, with an IC50 of 2.59 mg/mL (28). Wang has revealed free fatty acid receptor 2’s (FFAR2) critical role as a host factor in the replication of multiple IAV subtypes. FFAR2 deficiency significantly suppresses the replication of diverse IAV subtypes, including H1N1, H5N1, and H9N2, in the lung cancer cell line A549 and the mouse macrophage cell line RAW 264.7, and consequently decreases viral titers by as much as several dozen-fold. Furthermore, the FFAR2 agonists 4-CMTB and Cmp58 also exhibit dose-dependent inhibition of viral replication. Crucially, FFAR2’s supportive role is specific to IAVs and has no effect on the replication of other viruses such as vesicular stomatitis virus (VSV). Therefore, FFAR2 is a critical host target for influenza A viral replication with potential for antiviral therapeutic applications. Mechanistic studies have revealed that FFAR2 participates primarily in the early stages of IAV virus infection, particularly during viral endocytosis. Although FFAR2 does not affect viral binding to sialic acid receptors on the cell surface, its knockdown significantly impedes viral endocytosis and the accumulation of NP protein in the nucleus, thereby inhibiting viral entry. FFAR2 promotes viral endocytosis through direct interaction with viral HA1 and M2 proteins. Furthermore, FFAR2-mediated viral endocytosis relies on the synergistic action of β-arrestin1 and the AP-2 complex. G protein-coupled receptor kinases (GRK2, GRK5, and GRK6) enhance FFAR2 phosphorylation, thereby promoting its binding to β-arrestin1 and amplifying the clindamycin-dependent endocytic pathway for IAV. These findings have not only advanced understanding of influenza viral entry mechanisms but also provided a theoretical basis for developing novel anti-influenza strategies targeting the FFAR2 pathway (50).

Reading et al. have revealed significant differences in the efficiency of three IAV strains (BJx109, HKx31, and A/PR8 (H1N1)) in infecting mouse macrophages: BJx109 exhibits the highest infectivity, and is followed by HKx31, whereas PR8 demonstrates the lowest efficiency. These differences in efficiency correlate primarily with the glycosylation level of the viral HA protein: more highly glycosylated strains are more readily recognized and infected by macrophages. Further experiments have demonstrated that the mannose receptor (MR) on macrophage surfaces mediates viral binding and infection by recognizing high-mannose glycans on viral HA. Moreover, MR expression levels significantly influence viral infection rates. Therefore, MR, a key recognition molecule on host cell surfaces, plays a crucial role in IAV infection of macrophages. The interaction between the virus and MR might influence the host immune response, such that viral infection relies on the synergistic action of MR-mediated glycan recognition and sialic acid receptors. These findings provide new insights into the interaction between IAVs and immune cells, and aid in the development of novel antiviral strategies based on blocking virus-receptor interactions. They therefore might have potential clinical applications in regulating immune cell infection and inflammatory responses (51).

##### 3.3.1.2 Inhibition of viral replication

IAVs replicate within the cytoplasm of host cells by using host cellular materials and systems to complete reverse transcription of RNA and synthesis of the NP. During this phase, viral particles proliferate extensively while evading immune recognition. Once released, these particles trigger severe inflammatory responses and physiological dysfunction. TCM can inhibit viral proliferation by targeting multiple sites and pathways during replication. It interferes with early viral replication and binds viral NP, thereby restricting the nuclear export and oligomerization of viral NP (38), and ultimately interrupting the viral invasion process.

In research on the anti-influenza virus mechanisms of TCM, Zhou et al. discovered that Astragalus decoction is rich in the plant microRNA molecule MIR2911. This molecule remains highly stable during decoction, can be absorbed by the mouse intestine after oral administration, and is effectively delivered to lung tissue. MIR2911 directly targets key genes of multiple IAVs (including H1N1, H3N2, H5N1, and H7N9). By suppressing expression of the viral proteins polymerase beta 2 (PB2) and non-structural protein 1 (NS1), MIR2911 significantly decreases viral replication efficiency and viral load, mitigates infection-induced weight loss, and increases survival rates in infected mice (39).

In contrast, research by Guan et al. has revealed that pterodontic acid exhibits significant inhibitory activity against multiple IAV subtypes. Pterodontic acid not only mildly suppresses the neuraminidase activity of certain viruses but also suppresses viral replication by blocking the transport of viral RNP from the nucleus to the cytoplasm via inhibition of NF-κB signaling pathway activation. Furthermore, pterodontic acid markedly decreases the excessive inflammatory response induced by IAV infection. This molecule suppresses the expression of multiple pro-inflammatory cytokines and chemokines and consequently mitigates cytokine storm phenomena (40). Similarly, Coptis chinensis water extract exhibits viral replication suppression by inhibiting IAV RNA-dependent RNA polymerase activity without causing host cell toxicity (52).

To address novel viral pathogens, Huang et al. have conducted a systematic evaluation of the clinical efficacy of six traditional Chinese medicinal formulations (Jinhua Qinggan granules, Lianhua Qingwen capsules, Qingfei Paidu decoction, Xuanfei Baidu granules, Huashi Baidu granules, and Xuebijing Injection) for COVID-19 treatment. Clinical trials and randomized controlled studies have demonstrated that these formulations significantly alleviate clinical symptoms, enhance patient recovery rates, shorten disease duration, and decrease mortality. Active constituents in these formulations, such as quercetin, flavonoids, baicalin, and glycyrrhizic acid, target key SARS-CoV-2 proteins (including the angiotensin-converting enzyme 2 (ACE2) receptor and 3C-like protease (3CL) protease) and the inflammatory cytokine IL-6. This action inhibits viral replication and invasion while modulating the host’s immune and inflammatory responses (23).

Sun et al. have systematically evaluated the antiviral activity of carnosol against IAV and its mechanism of action, and revealed its significant potential for clinical application. The underlying mechanisms include direct disruption of the viral envelope and subsequent inactivation, as well as suppression of protein and mRNA expression during early stages of the viral life cycle. Furthermore, carnosol’s antiviral efficacy is enhanced by blocking viral replication through inhibition of the host cell the Janus kinase 2/signal transducer and activator of transcription 3 (Jak2/STAT3) signaling pathway. In animal models, oral administration of carnosol significantly increases survival rates in influenza-infected mice, decreases pulmonary viral load, and mitigates lung tissue pathology, with efficacy comparable to that of oseltamivir (41). Furthermore, studies on the TCM formula Artemisia annua granules (CYZH) have revealed broad-spectrum antiviral activity against multiple IAV strains *in vitro*, including IAV and influenza B viruses, as well as oseltamivir- and amantadine-resistant strains. CYZH exhibits dose-dependent inhibition of viral M2 protein expression and RNA replication, and significantly decreases viral replication levels. Notably, CYZH does not directly inhibit IAV HA or RNA-dependent RNA polymerase. Further mechanistic studies have revealed that CYZH significantly upregulates the mRNA and protein expression of the antioxidant enzyme heme oxygenase-1 (HO-1), in a manner dependent on activation of the nuclear factor erythroid 2-related factor 2 (Nrf2-ARE) signaling pathway. CYZH significantly suppresses virus-induced reactive oxygen species production, mitigates cellular oxidative damage, and decreases virus-induced cytopathic effects (31).

#### 3.3.2 Modulation of host inflammatory pathways and expression of inflammatory factors

IAV primarily attacks the respiratory tract, and causes swelling of the airways, lung inflammation, and infiltration of inflammatory exudate into the alveoli. The massive expression of pro-inflammatory cytokines and activation of signaling pathways involving TLR7, MyD88, Interleukin-1 receptor-associated kinase 4 (IRAK4), and NF-κB collectively induce inflammatory damage to the lungs. Consequent symptoms, such as respiratory distress and pulmonary edema, can be fatal in severe cases. During massive and rapid expression of inflammatory cytokines, immune system disruption leads to an imbalance in immune cell ratios that cannot be rapidly restored through endogenous mechanisms. Because TCM has both anti-inflammatory properties and an ability to modulate inflammatory pathways, it can effectively protect the host against virus-induced inflammatory damage. Forty-seven proto-extrinsic compounds and 13 metabolites were detected in rat plasma. Network analysis identified 145 overlapping targets associated with the Gene Ontology (GO) and Kyoto Encyclopedia of Genes and Genomes (KEGG) pathways for antipyresis and anti-inflammation. Molecular docking revealed binding energies below for key bioactive compounds—asprerologin B, vebascoside, isofobascoside, chlorogenic acid, and 4-O-lignylated pyridine. Core proteins included phosphatidylinositol-4,5-bisphosphate 3-kinase catalytic subunit alpha (PIK3CA), AKT1, NF-κB, and inhibitor of NF-κB alpha (IκB-α). Shiqi Waigan Granules significantly reduced LPS-induced fever in rats, an effect likely mediated by the inhibition of pro-inflammatory cytokine production (32).

Huanglian Xiangru decoction has been found to significantly decrease serum levels of IL-6, TNF-α, IFN-γ, and NO in infected mice; markedly increase IL-2, superoxide dismutase (SOD), and GSH levels; and substantially decrease TLR3, TLR7, MyD88, TNF receptor associated factor 3 (TRAF3), NF-κB, and p65 mRNA and protein expression in infected mouse lung tissue. This treatment inhibits the expression of inflammatory factors and the activation of inflammatory pathways in mice, thereby counteracting the effects of IAV (33). Lanqin oral liquid decreases inflammatory factor levels in bronchoalveolar lavage fluid by decreasing alveolar macrophages and monocyte-derived macrophages, and modulating the TLR4-PI3K-AKT-NF-κB signaling pathway, thereby treating H1N1 PR8-induced acute lung injury (ALI) (53). The traditional formula “HQQDT” contains baicalein, a NA inhibitor, which is a potent neuraminidase inhibitor and regulates inflammatory responses through the JAK-STAT signaling pathway (42). The patented drug Qingjie Tulei Granules (derived from the famous anti-influenza medicine formula Shengjiang San) can regulate the signal transducer and activator of transcription 1/3 (STAT1/3) signaling pathway and contains curcumin and curcumin derivatives, which inhibit HA (54) and NA activity (55, 56).

In mice with PR8-induced ALI, the baicalin glycoside complex (FESR) and its major constituents in Lanqin oral liquid, after biotransformation by the gut microbiota, exist primarily as aglycone forms, whose levels are significantly elevated under pathological conditions. Aglycone components (such as baicalin and baicalein) dose-dependently restore mTOR phosphorylation levels, inhibit PR8-induced autophagosome formation, decrease viral replication, effectively mitigate lung injury, decrease the lung index and lesion area, and suppress pulmonary complement deposition. Consequently, they protect against IAV-induced ALI with superior efficacy to that of aglycone precursors and the FESR complex (43). H3N2 induces autophagy by inhibiting the mTOR signaling pathway, thereby promoting its own replication and proliferation. Zhu has confirmed that under *in vitro* conditions, baicalin dose-dependently suppresses the expression of the Atg5-Atg12 complex and LC3-II in H3N2-infected A549 and Ana-1 cells; mitigates autophagic phenomena; and consequently inhibits viral replication (44). Sophocarpine has been found to decrease the mRNA and protein expression of retinoic acid-inducible gene I (RIG-I), NF-κB, and mitochondrial antiviral signaling (MAVS) in infected mice by inhibiting NLRP3 inflammasome activation, thereby downregulating GSDMD expression and alleviating pneumonia inflammation (45, 57). Atractylenolide-III inhibits IAV infection and virus-induced inflammation by binding viral NS1; modulating macrophage polarization and consequently decreasing proinflammatory signaling activation; and disrupting the NS1-CPSF4 interaction, which mediates selective polyadenylation (58). Huang’s previous research supported the virus clearance function of senna leaves (GLY) (59). Seven anti-influenza phytochemicals were isolated and their target proteins were identified. Canarone glycosides A and C, urolithin M5, mycelialin, and kemerol inhibited NA activity, while canarone glycoside B and quercetin targeted HA (56). In addition, GLY contains the PB2 inhibitor compound D715–2441 and methyl brevicol carboxylate, as well as the HA inhibitor isoflavone (60, 61).

Wei et al. have revealed that baicalin, the primary component of Scutellaria baicalensis, enhances the survival rate of alveolar epithelial cells infected with the IAV. This mechanism involves inhibition of virus-induced pyroptosis by blocking the caspase-3/gasdermin E pathway (62). Zhou et al. have demonstrated that the natural polyphenol pyrocatechol activates Nrf2 and PPAR-γ, thus promoting their nuclear translocation and synergistically enhancing HO-1 expression. Subsequent inhibition of the JAK1/STAT pathway independently of HO-1, and RIG-I-enhanced STAT1/2 activation dependent on HO-1, ultimately block the transcriptional activity of the ISGF3 complex. This action effectively suppresses the excessive inflammatory response and cell death induced by H1N1 virus in IFN-β-sensitized cells and therefore has potential as an anti-influenza agent (63).

The aforementioned research has revealed that certain key active constituents in TCM exert distinct mechanisms of action and have potential therapeutic value in inhibiting IAV replication and mitigating pulmonary tissue damage by modulating crucial cell death and inflammatory signaling pathways.

#### 3.3.3 Correcting metabolic dysregulation through the lung-gut axis

In TCM, the lungs and large intestine are considered to be paired as “interior-exterior” organs and to have a close relationship. Modern medicine also approaches respiratory diseases by considering the lung-gut axis, according to the principle of treating upper ailments by addressing lower conditions. This approach focuses primarily on regulating the diversity and species composition of the gut microbiota to treat pneumonia caused by the IAV.

According to modern medical research, in mice infected with IAV, mRNA expression of the H1N1 M gene is upregulated in lung tissue, and the relative expression of ROR-γ mRNA in intestinal tissue is also elevated. Concurrently, the relative expression levels of the cytokines GM-CSF and IL-2 in intestinal tissue are significantly elevated, thus demonstrating that H1N1 can induce pneumonia-enteritis symptoms (64).

The bacterial species in the large intestine are broadly classified into phyla including Firmicutes, Bacteroidetes, Actinobacteria, and Proteobacteria. The acidic polysaccharide (ephedra polysaccharide ESP-B4) in anti-H1N1 Chinese herbal medicine has been found to influence the structure and function of the intestinal microbiota, by increasing the abundance of beneficial gut bacteria; significantly increasing functional enzyme activity; and enhancing cellular activity states such as transport and catabolism, replication and repair, and nucleotide metabolism abundance. Chinese herbal medicine not only affects the state of the large intestinal microbiota but also positively influences the mucosal immune function of the small intestine (34). Bu Zhong Yi Qi Tang contains multiple low-molecular-weight hydrophobic components, along with at least one neutral component and three weakly acidic or strongly acidic polysaccharides. These hydrophobic components and polysaccharides influence the immune function of T lymphocytes within Peyer’s patches in the ileum, thereby promoting production of IFN-γ, IL-2, and IL-4. In summary, the multifaceted constituents within Bu Zhong Yi Qi Tang regulate the lower respiratory tract immune system through synergistic modulation of immune-active cells within the ileal region (29).

#### 3.3.4 TCM immunological adjuvants modulating immune signaling pathways

Beyond the three conventional antiviral approaches described above, new therapies are being explored from the perspectives of immune adjuvants and immune signaling pathways. Efforts are underway to integrate traditional medicine with modern medicine, and consequently combat the IAV more conveniently and effectively.

In modern medicine, standard treatments for influenza A include vaccination or antiviral medications such as oseltamivir, ribavirin, and peramivir. The trivalent inactivated vaccine and intranasal live attenuated influenza vaccine are the two most common influenza vaccines currently available. Trivalent inactivated vaccine often causes injection site swelling and has uncertain protective efficacy, whereas live attenuated influenza vaccine is unsuitable for pregnant individuals, and people with chronic illnesses or severe egg allergies (65). Additionally, IAV RNA is highly unstable, lacks proofreading mechanisms, and is prone to antigenic drift or shift (29, 65–68). Repeated vaccination can decrease vaccine efficacy in subsequent years and lead to immunological failure. Antiviral drugs can cause immune suppression and drug resistance (69), or mild self-limiting adverse reactions such as gastroenteritis (70).

A novel H3N2 vaccine using polysaccharides as a nanocarrier, conjugated with inactivated H3N2 virus and astragaloside VII (AST-VII), enhances IgG1 antibody titers by inducing IFN-γ, IL-17A, and IgG2 production while modulating Th1/Th2 balance and Th17a levels (71). Xiaoqinglong decoction exhibits oral adjuvant activity for intranasal influenza vaccines. Oral administration of its extract, pinellic acid, to mice receiving single and double intranasal inoculations of influenza A vaccine has been found to enhance antiviral IgA antibody titers in nasal and bronchoalveolar lavage fluids (72). The herbal adjuvants of Corydalis yanhusuo and andrographolide with influenza vaccine in mice have been found to enhance influenza vaccine-induced hemagglutination inhibition antibody titers, splenic cell proliferation, splenic T-cell differentiation, bone marrow dendritic cell maturation, and Th1/Th2 cytokine secretion to varying degrees. This treatment also avoids excessive inflammatory responses while simultaneously exerting bidirectional regulatory effects (68).

Because H1N1 enhances glycolysis and hypoxia-inducible factors 1 (HIF-1) signaling pathways in alveolar epithelial cells, inhibiting glycolysis or the HIF-1 pathway might provide novel therapeutic approaches for treating influenza A (73). Bai Gao Yichun, an alkaloid in Banlangen (Isatis root), elevates MAVS protein expression by decreasing mitofusin-2 expression. Subsequent increases in IFN-β and interferon-induced transmembrane protein 3 (IFITM3) ultimately suppress stress-induced IAV infection (46).

#### 3.3.5 Synergistic effects between TCM and other drugs

The combination of TCM with existing antiviral drugs is a notable direction in modern medicine that offers unique value in the comprehensive treatment of influenza. TCM formulations, leveraging their multi-component, multi-target synergistic effects, have substantial advantages in treating influenza A: they can directly inhibit viral replication while also modulating immunity and mitigating damage, and they have synergistic potential in combination with existing antiviral drugs. For example, synergistic effects of baicalin combined with ribavirin have been observed by Chen et al. in studies evaluating their combined efficacy against H1N1. *In vitro* experiments have demonstrated that baicalin 0.125 μg/mL + ribavirin 12.5 μg/mL significantly synergistically inhibit viral replication, increase cell survival rates, and decrease viral M protein expression. In mouse models, the combination significantly increases survival rates, mitigates weight loss, and alleviates pulmonary inflammation. This synergy, arising from distinct mechanisms, enhances therapeutic efficacy while potentially decreasing ribavirin dosage and adverse effects, and therefore has considerable clinical potential (74).

Further research has focused on LH, a TCM compound widely used for influenza treatment. Given the increasing risk of drug-resistant IAVs, developing new drugs and exploring combination therapies of LH with existing antivirals (such as oseltamivir/Osel or baloxavir marboxil/Bal) is important. Zhang et al. have systematically evaluated the effects of LH alone and in combination with Osel/Bal against seasonal IAVs (IAV/IBV) in both *in vitro* and *in vivo* models. In an H1N1-infected mouse model, LH monotherapy decreased weight loss and improved survival rates; whereas the combination of LH with Osel or Bal more significantly decreased intrapulmonary viral titers, more effectively alleviated pulmonary pathological damage, and decreased inflammatory factor expression. Transcriptomic analysis revealed that the LH+Osel/Bal combination significantly upregulated the expression of genes associated with antiviral and anti-inflammatory effects (75).

Additionally, Haidari has discovered that pomegranate extract (PPE) significantly inhibits the proliferation of IAVs such as H3N2, H1N1, and influenza B. PPE not only has direct virus-inactivating capabilities but also suppresses the viral RNA replication process and consequently decreases viral replication efficiency within cells. The primary active component of PPE, punicalagin, has significant efficacy in inhibiting viral replication and red blood cell agglutination. When combined with the existing antiviral drug oseltamivir, PPE exhibits synergistic effects, by significantly enhancing oseltamivir’s inhibition of viral release. This synergistic interaction might not only decrease the required dosage of oseltamivir and the risk of drug resistance but also offer a novel combination therapy strategy for clinical influenza treatment with broad application prospects (76).

The combined application of TCM with existing antiviral drugs has considerable promise, particularly in influenza treatment, in which complementary advantages may be achieved. Future research should further examine their synergistic mechanisms to develop more effective therapeutic approaches.

### 3.4 Application of TCM in severe pneumonia

Viral pneumonia, caused primarily by influenza viruses, coronaviruses, and other respiratory pathogens, is characterized by direct damage to the alveolar epithelium and an excessive immune response, which together lead to severe inflammation and oxidative stress. In critical cases, it can also result in acute respiratory distress syndrome and multiple organ failure (76).

For patients with severe influenza and those at high risk of severe disease despite mild symptoms, Wu’s study recommends Lianhua Qingwen capsules in combination with antiviral drugs and supportive therapy. Although evidence is limited, this regimen might have clinical value for widespread use, given the poor prognosis of severe influenza (77). After administration of Jiwei Qianghuo decoction combined with Zhuyeshi Gao decoction, NF-κB-mediated TNF-α signaling pathways and IFN-γ responses have been found to be downregulated in patients with fatal influenza A pneumonia, thus significantly inhibiting the production of various proinflammatory cytokines and chemokines (78). Li has established an ALI mouse model infected with the IAV strain H1N1 (A/PR/8/34). Treatment with Compound Double Flower oral liquid has been found to effectively restore antiviral immunity in mice by modulating protein expression levels in the TNF-mediated IFN signaling pathway and NLRP3 inflammatory signaling pathway (79).

The combination of Re Duan Ning Injection, Xi Yan Ping injection, Tan Re Qing injection, and Xue Bi Jing injection with conventional drug therapy for older patients with severe pneumonia has been found to increase clinical efficacy, effectively decrease white blood cell counts and C-reactive protein levels, and lower procalcitonin levels (80). Wang has evaluated the efficacy and safety of Chinese medicine versus Western medicine in patients with early-stage severe COVID-19. Chinese medicine achieved favorable antiviral effects and promoted the resolution of pulmonary lesions in patients with severe COVID-19, while effectively decreasing inflammatory responses. Moreover, its safety profile was comparable to that of Western medical treatment (81). Houttuynia cordata has been found to promote resistance to sepsis and lethal pneumonia caused by multidrug-resistant Acinetobacter baumannii infection in 5-fluorouracil-treated mice via the mTORC1-IFN-I signaling pathway (82). In mouse models of acute pneumonia, 97 components of Qingfei Paidu granules in the serum and lung tissue have been associated with 350 pneumonia-associated targets and key pathways. Qingfei Paidu granules alleviate ALI and inflammation in mice with lipopolysaccharide-induced acute pneumonia, thus decreasing monocyte, neutrophil, lymphocyte, and leukocyte counts in bronchoalveolar lavage fluid, while also decreasing TNF-α and IL-1β levels (83). Glycyrrhizic acid has been found to alleviate lipopolysaccharide-induced WI-38 fibroblast cytotoxicity, inflammation, oxidative stress, and ferroptosis via methyltransferase-like 14 (METTL14) knockdown (84).

Traditional Kampo medicines (TKMs) with a heat-clearing effect have anti-inflammatory effects, such as a reduced NF-κB activity, After oral administration, TKMs interact with the gut microbiota, producing two metabolites: metabolites from the gut microbiota (both food and host-derived), and TKM compounds converted from the gut microbiota. Both metabolites reduce the levels of pro-inflammatory cytokines (85). Lianhua Qingwen granules (86), Jiawei Yupingfeng powder (87), Toujie Quwen granules, and Buzhong Yiqi decoction decreased the levels of erythrocyte sedimentation rate (ESR) or C-reactive protein (CRP), and increased lymphocyte count; Lianhua Qingwen granules (86) reduced the level of D-dimer and increased the levels of albumin and hemoglobin; Qingfei Paidu decoction (88) reduced the levels of CRP, creatine kinase (CK), creatine kinase-myocardial band (CK-MB), lactate dehydrogenase (LDH), and blood urea nitrogen (BUN); Maxing Shigan decoction decreased the levels of CRP, IL-6, alanine aminotransferase (ALT), aspartate aminotransferase (AST), and serum creatinine (Scr), and increased CD4^+^ T and CD8^+^ T cell counts; and Tanreqing capsule (89) increased CD3^+^ T cell counts.

### 3.5 New explorations of the mechanisms of TCM therapies against influenza A

Currently, the holistic regulatory concept of TCM still lacks a modern molecular biological theoretical basis. With the advancement of network pharmacology and through computer-aided virtual screening of active TCM components against IAV, compounds possessing large hydrophobic groups capable of forming strong hydrophobic interactions and additional hydrogen bonds with the viral NS1A protein have been found to exhibit superior inhibitory effects (75). New derivatives of epi-cinnamaldehyde and rosemary have demonstrated simultaneous inhibition of both HA and NA, with diminished susceptibility to drug resistance (76). Hu has identified 242 bioactive components in the Ma Xing Shi Gan decoction through ultra performance liquid chromatography-high resolution mass spectrometry (UPLC-HRMS) and analyzed 56 of them with network pharmacology techniques, thereby revealing 338 targets and 99 core proteins in the protein-protein interaction network associated with ALI induced by IAV (90, 94). Xue et al., through use of gene chips, have discovered that multiple inflammatory factors and chemokines show elevated gene expression in the lungs of mice infected with H1N1 PR8, and activation of various inflammatory signaling pathways. The lung tissue exhibited elevated levels of two complement proteins, CD4 and CD62P, and miRNA target genes were identified as potential new therapeutic targets (91). Ji has used a combination of novel nanocapsules, 3D-printed microfluidic chips, and smartphones to monitor the H7N9 avian influenza pathogen, and achieved detection performance comparable to that of traditional ELISA methods (92). Li has analyzed biomolecular networks based on clinical transcriptomics to evaluate key mechanisms underlying IAV infection. With UHPLC-Q-Exactive-Orbitrap-MS/MS systems, sample components in mouse serum were identified, and Compound Double Flower oral liquid was demonstrated to rebalance antiviral homeostasis in influenza pathogenesis by inhibiting TNF-mediated IFN/NLRP3 inflammasome activation (34). Moreover, Liu has integrated large-scale and single-cell RNA sequencing datasets to dissect pan-apoptotic cellular heterogeneity and transcriptional dynamics in IAV-infected lungs (93).

### 3.6 Challenges for TCM anti-influenza mechanism studies

Based on the above review, existing evidence for the anti-influenza effects of TCM mainly comes from animal and cell studies. TCM can inhibit various processes of viral adhesion, invasion, and replication, but the molecular mechanisms of its antiviral effects at different stages are still unclear. TCM’s anti-inflammatory mechanisms are primarily studied through inflammation-related signaling pathways (such as the NF-κB and Nrf2 pathways) or cellular proteins (such as MIP-1β, IL-8, and IP-10), however, these molecular insights remain relatively superficial, leaving many gaps in our understanding. Overall, current evidence on the anti-IAV mechanisms of TCM is constrained by several key limitations: reliance on oversimplified models, small study scales, complexity of pathway interpretations, and unclear dose-response relationships. Specifically, the use of cell lines and inbred mouse models fails to replicate the complex immune environment and inter-organ interactions in humans, necessitating caution when extrapolating findings to clinical settings. Most studies remain exploratory, conducted by single laboratories with limited sample sizes, and lack large-scale, multi-center validation. Moreover, inflammatory signaling networks are highly redundant and interactive; thus, targeting only one or a few pathways or factors is insufficient to account for the holistic anti-inflammatory effects of TCM formulas. Many studies report outcomes only at specific doses without systematic dose-gradient experiments, making it difficult to establish optimal therapeutic windows and undermining result reliability and reproducibility. Finally, publication bias may exist in this field, as studies reporting significant positive results are more likely to be published, whereas those with negative or inconclusive findings may remain unpublished. This could lead to a systematic overestimation of the anti-IAV efficacy of TCM.

As we know, the therapeutic efficacy of TCM in the treatment of influenza is well recognized. However, TCM therapies exhibit greater complexity in their origins and constituents, and are characterized by synergistic effects of multiple substances including both the original components of TCM and secondary metabolites generated during the exposure and metabolism of these substances within the body. The intrinsic complexity of TCM formulas—characterized by multi-component and multi-target interactions—poses significant challenges for elucidating their synergistic or antagonistic mechanisms within the framework of modern biomedical science. This complexity represents a major barrier to in-depth investigation of the molecular mechanisms underlying the antiviral effects of TCM. Moreover, TCM is fundamentally guided by the principle of syndrome differentiation and individualized treatment. In the context of viral infections, factors such as a patient’s physiological constitution and the presence of underlying diseases can substantially influence the selection and composition of herbal prescriptions. This inherent variability presents a critical challenge for achieving uniformity and consistency in clinical research data. In contrast to animal and *in vitro* experiments, which rely on highly standardized interventions, TCM clinical practice emphasizes personalized therapeutic strategies. Fixing prescriptions rigidly may compromise the core advantages of syndrome-based treatment, whereas allowing flexible modifications based on individual conditions inevitably introduces substantial heterogeneity, thereby complicating statistical analysis and limiting the ability to derive unified conclusions. This dilemma is one of the fundamental reasons why high-quality evidence supporting TCM is still insufficient for strong recommendations in leading international clinical guidelines.

Furthermore, a comprehensive review of the literature on TCM for influenza prevention and treatment highlights a central challenge in the modernization of TCM: the inherent tension between individualized therapy and the demand for standardized evidence. Future breakthroughs may not depend on forcing TCM to conform entirely to conventional evaluation frameworks established for modern medicine, but in developing a new research paradigm suitable for “complex interventions”.

## 4 Conclusion and perspectives

IAVs frequently cross species barriers and threaten public health, while conventional antivirals and vaccines suffer from drug resistance and side effects. In contrast to conventional antiviral drugs that typically target a single viral component, TCM formulations and their active constituents exert multi-faceted effects by intervening at multiple stages of the influenza virus life cycle while simultaneously modulating host immune responses. TCM not only directly inhibits viral replication but also enhances immune defense mechanisms and mitigates immunopathology, thereby offering a dual regulatory advantage. Moreover, TCM components exhibit significant synergistic effects when used in combination with standard antiviral agents or vaccines, potentiating therapeutic efficacy and potentially reducing the risk of drug resistance. A distinctive feature of TCM is its regulation of the gut microbiota to influence respiratory immunity, a concept grounded in the classical TCM theory of “lung and large intestine being interior-exteriorly related” and increasingly supported by modern scientific evidence. In the context of severe influenza, often characterized by excessive inflammation and cytokine storm, TCM has demonstrated potent anti-inflammatory and organ-protective properties. Its natural origin and multi-component synergy contribute to a favorable safety profile and low resistance potential, making it suitable for both therapeutic and prophylactic applications.

However, the anti-influenza effects of TCM are supported mainly by animal and cell studies, and researches are limited by overfocus on main components, failing to reflect TCM’s holistic characteristics. The intrinsic complexity of TCM formulas—featuring multi-component, multi-target interactions and individualized, syndrome-based treatment—creates a fundamental tension with the standardized evidence requirements of modern biomedical science. Future breakthroughs may require developing new research paradigms suited to complex interventions, rather than forcing TCM into conventional evaluation frameworks. In any case, high-quality evidence-based clinical research in medicine is not only the gold standard for verifying the efficacy of TCM, but also a bridge connecting “clinical effectiveness” and “clear mechanism”, playing an irreplaceable role in breaking through the core bottlenecks faced by the modernization of TCM. The long history of clinical use provides a rich knowledge base for modern drug discovery and mechanistic research, effectively bridging traditional wisdom with cutting-edge science.
